# A root-cause analysis of maternal deaths in Botswana: towards developing a culture of patient safety and quality improvement

**DOI:** 10.1186/1471-2393-14-231

**Published:** 2014-07-16

**Authors:** Farai D Madzimbamuto, Sunanda C Ray, Keitshokile D Mogobe, Doreen Ramogola-Masire, Raina Phillips, Miriam Haverkamp, Mosidi Mokotedi, Mpho Motana

**Affiliations:** 1 School of Medicine University of Botswana, Gaborone, Botswana; 2 Department of Anaesthesia and Critical Care Medicine, University of Zimbabwe College of Health Sciences, Mazowe Street, Belgravia, Harare, Zimbabwe; 3 School of Nursing University of Botswana, Gaborone, Botswana; 4 Government of Botswana/University of Pennsylvania [Botswana-UPenn] Partnership, Gaborone, Botswana; 5 Medical Education Partnership Initiative [MEPI] Maternal Mortality Project, Gaborone, Botswana

**Keywords:** Root-cause analysis, Maternal mortality, Contributory factors, Botswana

## Abstract

**Background:**

In 2007, 95% of women in Botswana delivered in health facilities with 73% attending at least 4 antenatal care visits. HIV-prevalence in pregnant women was 28.7%. The maternal mortality ratio in 2010 was 163 deaths per 100 000 live births versus the government target of 130 for that year, indicating that the Millennium Development Goal 5 was unlikely to be met. A root-cause analysis was carried out with the aim of determining the underlying causes of maternal deaths reported in 2010, to categorise contributory factors and to prioritise appropriate interventions based on the identified causes, to prevent further deaths.

**Methods:**

Case-notes for maternal deaths were reviewed by a panel of five clinicians, initially independently then discussed together to achieve consensus on assigning contributory factors, cause of death and whether each death was avoidable or not at presentation to hospital. Factors contributing to maternal deaths were categorised into organisational/management, personnel, technology/equipment/supplies, environment and barriers to accessing healthcare.

**Results:**

Fifty-six case notes were available for review from 82 deaths notified in 2010, with 0–4 contributory factors in 19 deaths, 5–9 in 27deaths and 9–14 in nine. The cause of death in one case was not ascertainable since the notes were incomplete. The high number of contributory factors demonstrates poor quality of care even where deaths were not avoidable: 14/23 (61%) of direct deaths were considered avoidable compared to 12/32 (38%) indirect deaths. Highest ranking categories were: failure to recognise seriousness of patients’ condition (71% of cases); lack of knowledge (67%); failure to follow recommended practice (53%); lack of or failure to implement policies, protocols and guidelines (44%); and poor organisational arrangements (35%). Half the deaths had some barrier to accessing health services.

**Conclusions:**

Root-cause analysis demonstrates the interactions between patients, health professionals and health system in generating adverse outcomes for patients. The lessons provided indicate where training of undergraduate and postgraduate medical, midwifery and nursing students need to be intensified, with emphasis on evidence-based practice and adherence to protocols. Action plans and interventions aimed at changing the circumstances that led to maternal deaths can be implemented and re-evaluated.

## Background

Concerns to improve quality of health services in Africa have mainly focused on increasing workforce numbers. Making health professionals more effective in what they already do also deserves priority. Analysis of trends in maternal mortality ratios (MMR) may reveal weaknesses in health systems that lead to maternal deaths, to establish where changes can be made to improve outcomes, especially in resource-limited settings [1,2]. Development of a culture of patient safety with aligned risk-assessment techniques has been central to improving the quality of maternity services in higher income countries [3]. Techniques of investigating safety incidents in healthcare, adapted from industrial settings, include root cause analysis (RCA) to identify factors contributing to the safety incident (maternal deaths in this review) [3]. These factors are categorised as patient characteristics, task factors (for example lack of protocols), individual staff factors, work environment, team-working, and organisational or management factors [4]. The use of RCA as a method of continuous quality improvement provides opportunities to create a culture of patient-safety within which health professionals can be more effective in providing patient-centred care. In Australia, root cause analysis was used to improve work practices and patient safety, to facilitate teamwork and communication about patient care [5].

In Botswana, maternal deaths have been notified since 1998 by health facilities to the Ministry of Health (MOH), with confidential case conferences conducted quarterly by the National Maternal Mortality Audit Committee [6,7]. Despite these efforts, the MMR has not declined sufficiently to meet the 2015 Millennium Development Goal 5 target [8]. In 2010, there were 49,853 institutional live-births and 475 non-institutional live-births, with 82 maternal deaths reported to the MOH, giving an MMR of 163 per 100 000 live births, versus the government target of 130 for that year [9]. A case-record review was conducted of these deaths, from which clinical details and classification were published earlier [10]. However, a deeper analysis was necessary to prioritise contributory factors so that interventions to address these could be designed to have more impact. The objective of this study was to determine the root causes of maternal deaths in Botswana using an RCA framework modified from Farquhar et al. [1] and suggest appropriate interventions that address these causes.

### Study setting

Botswana, a middle income country in southern Africa, has a population of 2 million served by 2 referral hospitals (that also provide district functions for the populations of Gaborone and Francistown), 31 district and primary hospitals, and 263 clinics providing antenatal care (including 92 with facilities for deliveries). Over 95% of the Botswana population lives within 15 kilometres of a health facility [11]. On other maternal health indicators Botswana performs well: in 2007, 73% of women attended at least 4 antenatal care (ANC) visits while 95% of all reported deliveries occurred in health facilities. A high proportion of deliveries were attended by health professionals: 97% of deliveries in cities, towns and urban villages and 90% of deliveries in rural areas [11]. HIV prevalence in pregnant women was estimated at 28.7% in 2010 [12]; 94% of HIV-positive pregnant women who were eligible for antiretroviral (ARV) drugs according to the 2008 National HIV Treatment Guidelines, were receiving them [13].

## Methods

In 2010, 82 maternal deaths were notified to the MOH through the National Maternal Mortality Audit Committee. The case notes for these women were requested from each reporting health facility. The cause of death and contributory factors were independently reviewed for each case by 2 pairs of clinicians and an HIV specialist, then discussed together to achieve consensus, as reported separately [10]. The question “why” was asked to elicit underlying explanations for each of the factors. Details were entered into a data entry table with structured headings for RCA (Table 1). Table 2 gives an example of how the RCA for one case was done. The contributory factors that were derived by asking “why” for all the cases were allocated to the categories in the modified framework of Farquhar et al. (Table 3) for direct and indirect causes of maternal deaths. As demonstrated in the example given in Table 2, each case generated several contributory factors which were categorised as organisational/management, personnel, technology and so on. The clinician panel decided whether each death was avoidable or not using Geller’s definition of whether “action or inaction on the part of the health care provider, system or patient … may have caused or contributed” to the adverse outcome [14]. Whether a death was avoidable or not was determined by examining the events related to the final admission rather than the entire course of pregnancy, since every case had opportunities for prevention from first contact with the health service, including primary prevention of pregnancy and HIV.

**Table 1:** Data entry instrument – asking whys model of root cause analysis (RCA)

**Time line**	**Clinical data**	**Root causes and comments**
Antenatal care period	Summary of ANC record with notes on significant events	What was the earliest significant event?
		How did it occur? Why?
		What was the next failure?
		How did that occur? Why?
		Why was this not corrected?
Admission presentation	Indications for clinic or hospital admission.	What factors were related to ANC? How?
		What factors contributed to outcome? How?
	Summary of clinical record.	Why did they occur?
	Notes on significant events.	Why was this not corrected?
	Was the diagnosis correct?	
Death	Cause of death given in the notes [clinical or post-mortem]	Consensus on most probable cause of death. Was death avoidable? How?
Root cause analysis: adapted from National Patient Safety Agency [15]		
1. Patient characteristics: pre-existing or co-morbid medical conditions, physical limitations, language and communication barriers, cultural issues, social support needs that play a role.		
2. Task factors: What protocols and procedures are in place for labor and delivery, for use of analgesia, for dystocia, for C-sections? Are they safe? Are they practical? Are they effective? Are they consistently applied?		
3. Individual staff: How did the knowledge, skills, training, motivation, and health of patient’s providers affect her care?		
4. Team factors: How well do the various health care professionals involved in patient’s care work together? What is the nature of the communication? Are there hierarchies? What is the responsiveness of nursing supervisors or attending physicians? How easily can a team member ask for help or clarification?		
5. Work environment: Is the labor and delivery unit adequately staffed? What is the workload? What is the staffing level of experience, functionality of the equipment, quality of administrative support?		
6. Organizational and management factors: How do the values of the hospital translate into clinical practice? Do their standards and policies focus more on patient safety and quality of care, or volume and speed? Are management’s priorities patient- or provider-centered? Does senior leadership foster a culture of teamwork and safety or blame and shame?		
7. Possible solutions:		

**Table 2:** **Example based on an actual maternal death showing application of the *Asking Why *** **Root Cause Analysis (RCA) method**

**Cause of death**: post-partum haemorrhage (PPH) with death in the ambulance during transfer from primary hospital to next level district hospital.	
**Why was there failure to control post-partum bleeding 4 hours after birth?** (from last to first circumstance)	
1. The bleeding was not controlled – post-partum haemorrhage and resuscitation was inadequate.	
2. The seriousness of the patient’s condition was not recognised or acted upon.	
3. There was delay in identifying that the laceration to her cervix was severe and continuing to bleed.	
4. The delivery of the baby was not controlled leading to tears in posterior cervix.	
5. At the ANC clinic, staff failed to refer a high risk grand multiparous woman for management at a higher level hospital where blood transfusion was available in case of need.	
**Sequence of events: contributory factors: asking why**	**Interventions required to address the gaps/weaknesses in health system identified in this case**
Why was there inadequate resuscitation prior to transfer, including no blood transfusion?	1. Training on clinical skills and principles of resuscitation.
	2. Assessment that the training leads to improved practice (clinical audit) in future.
	3. Enquiry as to why blood was not transfused: if it was not available at the primary hospital, this was a higher indication for early transfer or referral for management.
Why was there a delay in detecting PPH? A laceration was sutured post delivery but a deep tear in the posterior cervix was initially missed, then the attempted repair was insufficient with blood loss of at least1 litre over 2 hours.	1. Supervision of management of high risk patients: need for high level of suspicion in grand multiparous woman who develops post-partum bleeding.
	2. Training in management of lacerations and tears following delivery, especially those with severe bleeding.
	3. Guideline for management of lacerations in high risk patients by the highest level of surgical skills available in that health facility.
Why did the delivery result in lacerations?	1. Training and assessment of proficiency in controlled delivery of baby by skilled birth attendants.
Why wasn’t her hypotension more aggressively managed? It dropped from 100/60 to 80/? over two hours or more. She was given 2 doses of oxytocin in 10 IU boluses. There was poor documentation of the patient’s clinical condition and actions taken.	1. Training in assessment of the seriously ill obstetric patient.
	2. Need for a protocol on the use of oxytocin in such cases since this may have contributed to her hypotension.
	3. Need for evaluation of clinical skills of the medical and nursing staff involved with provision of refresher training.
	4. Supervision of record-keeping and documentation, with training on competent documentation of the patient’s vital signs, clinical condition and the actions taken.
Why was the woman’s care provided at a primary hospital when she had multiple risk factors? Despite 6 ANC visits her risks were not anticipated.	1. Need for protocol on referral of grand multiparous woman to a higher level hospital due to risk of PPH.
	2. Training and supervision of risk assessment by ANC staff.
Patient characteristics: 36 years old, G5P4, HIV positive on ART. She stopped her oral contraception because she wanted to change to an injectable one which was out of stock.	1. Need for training in communication skills: she should have been advised to continue with oral contraception or barrier methods until her alternative preference available.
	2. Primary PMTCT of HIV: prevention of unintended pregnancy (abortion not permissible under Botswana law for contraceptive failure despite risk to mother).

**Table 3:** Factors contributing to maternal deaths in Botswana 2010 Contributory factors identified (multiple categories apply) N = 55

	**Direct**	**Indirect**	**Total**	**% cases**
*Organizational and/or management total*	(42)	(89)	47	84
1. Lack of, or failure to implement, policies, protocols, guidelines	8	16	24	44
2. Poor organizational arrangements of staff	0	19	19	35
3. Inadequate education and training	10	8	18	33
4. Poor team work	5	12	17	31
5. Delayed ordering investigations, access to test results or inaccurate results	4	12	16	29
6. Poor access to senior clinical staff	7	8	14	25
7. Inadequate systems/process for sharing clinical information between services: all HV positive	1	10	11	20
8. Failure or delay in emergency response	5	3	8	15
9. Delay in intervention or procedure eg C-section	2	1	3	5
10. Inadequate numbers of staff	UK	UK	UK	UK
*Personnel total*	(62)	(96)	49	88
11. Lack of recognition of complexity/seriousness of condition	14	25	39	71
12. Lack of knowledge and skills of staff (includes failure to maintain competence, making wrong diagnoses, lack of differential diagnoses leading to linear decision making)	12	25	37	67
13. Failure to offer or follow recommended best practice	11	18	29	53
14. Failure to seek help/supervision/consultation/delay in physician/ICU/anaesthetic consultation	9	10	19	35
15. Failure of communication between staff (entries in medical notes used to communicate between doctors and nurses)	5	11	16	29
16. Delayed emergency response by staff	8	4	12	22
17. Failure of communication of staff with patient or family	3	3	6	11
*Technology, equipment and supplies total*	(5)	(11)	14	27
18. Supplies (IV fluids, blood for transfusion, drugs etc.) out of stock	4	8	12	22
19. Non-availability, malfunction or failure of essential equipment	1	3	4	7
*Environment (1 case, 2%)*			1	2
20. Geography eg long distance transfer	1		1	2
*Barriers to accessing or engaging with care total*	(18)	(21)	29	53
21. Did not attend for ANC, only had one ANC visit, or late booking	8	12	20	37
22. Not eligible to access free care (non-citizens)	7	4	11	20
23. Lack of recognition of complexity/seriousness of condition by either woman or her family	1	4	5	9
24. Maternal learning disability		1	1	2
25. Cultural barriers (attended traditional healer first)	1		1	2
26. Social circumstances	1		1	2

*Adapted from Farquhar* et al. *2011*[1].

Ethics approval was obtained from the University of Botswana Research Ethics Committee and Ministry of Health as well as each hospital from which records were obtained.

### Definitions

The World Health Organisation defines maternal death as the death of a woman while pregnant or within 42 days of termination of pregnancy, from any cause related to or aggravated by the pregnancy or its management, but not from accidental or incidental causes [16]. Direct maternal deaths are those resulting from obstetric complications of the pregnant state. Indirect deaths are those resulting from previous existing disease, or disease that developed during pregnancy, not due to direct obstetric causes but aggravated by the physiologic effects of pregnancy [16]. The standard of documentation in the case notes should provide a complete and accurate record of the patient’s condition, investigations and treatment, with sufficient detail to provide an audit trail to permit investigation if and when required [17]. The notes were considered to have poor documentation when these standards were not met.

## Results

Of the 82 deaths reported in 2010, 58 case-notes were provided by health facilities for review and 24 case notes were missing. One death occurred in 2009 and one death resulted from a stab wound, so these two cases were removed from the list. Of the 24 missing case notes some limited information was available on 19: ten were deaths at the two referral hospitals and 9 deaths were at either primary or district hospitals. This is a similar distribution to the cases that were reviewed (37 at referral hospitals, 16 at primary or district hospitals, one at home and 2 with place of death unrecorded but with high likelihood of being at hospital). The review was done on 56 case-notes. The cause and circumstances of death for the 56^th^ case was not ascertainable since the notes were incomplete. Poor documentation was noted in 13/23 (57%) direct and 18/32 (56%) indirect deaths. Table 3 shows the contributory factors as per the framework used and the number of cases with each factor. Contributory factors were identified in 54 of 55 of cases, with insufficient information in the notes for the 55th case to attribute contributory factors. Most cases had multiple factors: 19 deaths had 0–4 contributory factors, 27 deaths had 5–9 and 9 deaths had 10–14 contributory factors. The case shown in Table 2 had 9 contributory factors.

Factors relating to organizational/management, personnel, or barriers to access and engagement were more frequent than factors relating to the environment or technology and equipment. The highest ranked personnel factors were lack of recognition of seriousness and complexity of the patients’ condition (39 cases, 71%), followed by lack of knowledge and skills of staff (37 cases, 67%) and thirdly, failure to offer or follow recommended best practice (29 cases, 53%). In some instances correct aggressive clinical management of a problem was defeated by the failures in the system. In one case with 6 contributory factors, the death resulted from shortages of drugs at clinic, hospital and Intensive Care Unit (ICU) levels, non-functioning equipment in ICU and delays in laboratory investigations.

The factor which ranked highest in the organisational category was the lack of, or failure to implement, policies, protocols or guidelines (24 cases, 44%). Ranked second was poor organizational arrangements of staff, for example not having joint management plans such as between medical and maternity services (19 cases, 35%). Joint management is where specialists meet together to discuss how the patient should be managed from the point of view of their own specialty but in collaboration with each other. All the cases in this category were of indirect causes (15 cases were HIV positive and 4 were HIV negative). Cases were complex with problems requiring obstetric, medical and critical care inputs. Inadequate systems for sharing clinical information for instance between ANC and infectious disease care (IDC) clinics were more important in indirect than direct causes of death.

Table 4 shows the classification of causes of maternal deaths with corresponding contributory factors and potentially avoidable deaths. The high number of contributory factors for both direct and indirect deaths demonstrates the poor quality of care even if deaths were not avoidable: 14/23 (61%) of direct deaths were considered avoidable compared to 12/32 (38%) indirect deaths. Twelve deaths (52%) of direct causes compared to 25 deaths (78%) of indirect causes were in HIV positive women (12 women with clinical AIDS). Women who died of indirect causes often presented in a very poor clinical condition at admission. Some cases stated to be unavoidable may have been avoided if the seriousness of their condition was identified at earlier stages in the pregnancy. Occasionally women known to be HIV-positive were recorded as having persistent headaches or chronic cough at the ANC clinic but were not assessed for meningitis or tuberculosis or referred for management, later dying of AIDS during pregnancy or postpartum period.

**Table 4:** Contributory factors for each cause of maternal death

***Cause of maternal death***	***No of cases (N = 55)***	Total no. of contributory factors present for that cause of death*	***Potentially avoidable deaths***
Total Direct deaths (42%)	23	22	14
Hypertension (Eclampsia)	5	16	3
**Haemorrhage**	**10**	**17**	
APH	1	9	0
Vagina/cervix trauma	2	11	2
Bleeding during or after C-section	2	11	1
Ectopic haemorrhage	1	8	1
Non-traumatic haemorrhage	2	8	0
Uterine trauma	2	8	2
**Pregnancy related sepsis**	**3**	**9**	
Chorioamnionitis	1	7	Unknown
Puerperal sepsis after NVD	1	4	1
Septic miscarriage	1	1	0
**General anaesthetic**	**2**	**6**	**2**
**Pulmonary embolus**	**2**	**8**	**2**
**Acute collapse**	**1**	**1**	**0**
Death at home – cause unknown	1	1	0
**Total Indirect deaths (58%)**	**32**	**24**	**12**
**HIV**	**19**	**21**	**7**
Pneumonia	3	12	2
TB	2	11	0
Meningitis	3	14	0
GI tract	3	13	3
Complications of ARVs (including one with IRIS**)	4	15	2
Other (multiple organ systems)	4	9	0
**Medical –Surgical**	**13**	**20**	**5**
Cardiac	7	17	1
Endocrine/metabolic	3	12	3
Other adverse effects of treatment	3	5	1

**IRIS immune reconstitution inflammatory syndrome.

*Each contributory factor is only counted once for each group so the number of factors for each category is not the sum of the individual cases.

## Discussion

This study was a retrospective case-record review, showing aggregated data. The identification of the root causes of the deaths relied on the completeness of the case records and the expertise of the clinicians conducting the reviews. It was not possible to identify retrospectively from case records whether staff numbers were adequate, what the work environment was like, or to what extent inadequate education and training contributed to poor quality of care. There was also limited information recorded on patient and family factors. Since 95% of reported deliveries took place in health facilities [11] the risk of under-reporting maternal deaths is low but could occur from misclassification such as with deaths from indirect causes in the postnatal period. Women who die at home have to be brought to a health facility for death registration so would be notified but perhaps not identified as maternal deaths, in early pregnancy for example. The RCA checklist is useful if adopted as a guide to enquiry for each woman’s death as it happens, while contributory factors are fresh in the minds of the healthcare team responsible. If enquiries are carried out with a “no blame no shame” approach and an emphasis on learning from mistakes, health facilities will be transformed into learning organisations, supportive of staff development.

Half the deaths in this review had some barrier to engaging with healthcare, either because patients were not eligible for free treatment as non-citizens, or did not attend sufficient antenatal care for their risks to be identified. “Free maternity services for all” requires policy changes alongside public education campaigns to inform non-citizens of their rights. Barriers to early attendance at antenatal clinics, especially in the context of HIV and prevention of mother to child transmission (PMTCT), must be addressed and antenatal screening for opportunistic infections such as tuberculosis encouraged. Pregnant women are given education on danger signs during antenatal care, but may not be in a fit state to alert their families to their risks. One woman had seizures for 24 hours while another woman suffered from decreased consciousness due to meningitis for several days, before their families took them to health facilities. Health education materials in local languages should target families as well as pregnant women.

The findings from this set of RCAs are similar to other enquiries into maternal deaths in the region and the experience of suboptimal use of protocols and guidelines in Botswana. A maternity services’ audit in Malawi found that poor documentation, delays in recognising the severity of the clinical condition, delays in adequate treatment and preoperative resuscitation with delays in referral, contributed to substandard care [18]. In Tanzania, 69% of maternal deaths were related to substandard care [19]. In Botswana, an audit of management of acute respiratory infections and diarrhoea in children revealed suboptimal adherence to guidelines on history-taking and poor clinical examination of cases [20]. Other studies in Botswana showed that only 30% of health professionals used the recommended dose of oxytocin at caesarean section [21] while National Guidelines on initiation of treatment for tuberculosis were not followed in 47% of cases [22].

This study revealed multiple weaknesses in the health system that led to maternal deaths. Occasional individual errors, unsafe acts by health professionals or single instances of system failures, may not threaten patient safety. However when they line up without protective measures in place, adverse events compound each other leading to serious incidents such as maternal deaths or “near misses”. Near- misses are those where the mother survives the incident of grave illness but may suffer disability or injury as a result. This chain of events will repeat itself if changes are not made to strengthen the system’s defences [2,23]. Single interventions or an isolated focus on human error are unlikely to impact significantly on maternal morbidity and mortality. Travis et al. explain that it is more common for operational constraints to have several underlying interdependent factors rather than a single root cause, with greater success in overcoming such constraints if these interdependent relations are accounted for [24].

This review shows that RCA methods are useful in medium and low-income contexts for prioritising interventions and generating action plans for achieving change, especially through more effective use of existing resources. Each RCA generates a list of factors with corresponding solutions or interventions, or suggests further enquiries such as why a particular protocol was not used. When aggregated as in Table 3, a quantifiable list of priorities emerges for development of action plans to address these issues, with immediate, medium term and long-term activities laid out including measurable outcomes and time limits (see Table 5). These action plans with standards and indicators can be re-audited to gauge progress made over a time period. Research has shown that health professionals are motivated to improve patient safety if given guidance through mentoring, supervision, training and support [5,25]. Introducing quality improvement as an integral part of undergraduate and postgraduate clinical curricula enables changes in values and attitudes that put patient safety high on the agenda. Medical and nursing training must reinforce the importance of good documentation of clinical records, team-work, communication and consultation skills. Trainees gain skills in critical appraisal, proposing changes based on their reviews and evaluating the impact of these changes as part of the audit/QI cycle. Mentorship, supportive supervision and constructive feedback are crucial in reinforcing confidence in trainees to recognise and manage serious complex conditions, including seeking early opinions from seniors, and must be strengthened throughout the teaching health system. Further research is necessary on staff attitudes towards their patients, why they did not communicate well with each other or with patients and their families, and why staff neglected to use protocols or guidelines. These audits provide the backbone for postgraduate dissertations, offering opportunities for publication in regional journals [21,26,27]. Detailing the difficulties in conducting audits is also necessary for removing obstacles [26]. Life-long learning methods, problem-solving approaches, development and use of early warning scores, regular drills on team responses to emergencies are evidence-based methods of addressing maternal deaths and part of the quality improvement repertoire.

**Table 5:** **Possible action plans arising from the example in Table**2

**Time frame**	**Type of intervention**
**Immediate (one-to-one guidance and supportive supervision)**	• Identify who is in charge of quality assurance in midwifery at all health facilities and will take the lead on actions recommended
	• Ensure immediate supervisory visits include aspects of proficiency in risk identification and assessment in ANC, controlled vaginal delivery, post-delivery examination of vagina and cervix for tears and injury, management of bleeding, resuscitation skills, recognition of seriously ill obstetric patients and when to act with urgency;
	• Check when the next Emergency Obstetric Care (EmOC) or similar training is due to take place and prioritize this, bringing it forward if possible;
	• Check that all facilities have protocols that include use of oxytocic agents, that they are using them, and if not, assess the barriers to use;
	• Check that there is a guideline on logistics management of daily availability of blood supplies as per facility level, whether this is being used, and assess barriers to use.
**Mid-term (training, drills, protocol review)**	• Review that protocols are up-to-date, in place and being used for use of oxytocic agents;
	• Organize drills in management of severe obstetric haemorrhage;
	• Organize consultations on communications between senior and junior level health professionals, doctors and nurses, on how to get more expert advice provided by mobile phone and email, joint ward rounds including senior staff, specialist outreach visits to peripheral facilities to train, guide, mentor, create more ownership over guidelines and protocols; facilitate closer senior supervision of management of cases with risk factors
	• Training and supervision of competent documentation and record keeping of clinical cases, vital signs and actions taken
	• Clinical audits of management of patients for example in risk assessment at ANC, compliance with national or local protocols for a variety of conditions, feedback and re-audit
	• Action to improve blood supply through mobilization of blood donors.
**Long-term (systemic curricula review; policy guidance; changing attitudes and practices)**	• Identify current competencies of staff against expected competencies for that level hospital, examine training curricula for relevance
	• Develop new protocols and policies, update with reference to national and international evidence of effectiveness including policies on blood transfusion and logistical supplies of blood at facility level.

The health-related Millennium Development Goals renewed focus on health system strengthening in medium and low-income countries, with calls for urgent investments in human resources, information systems, infrastructure, supplies, planning, management, supervision, and monitoring [28]. Quality improvement methods in countries including Ghana and Tanzania led to improving responsiveness to obstetric emergencies, referral systems, capacity-building within the health workforce and upgrading health centres in hard-to-reach areas [29-31]. Evidence on effective implementation of guidelines, protocols and policies include ownership and incorporation of local practical experience as key components [32]. Updating policies, protocols and guidelines as teams, while checking on their local relevance and applicability, is a good way of encouraging their use. The case studies developed through this and other QI programs are a valuable resource for focusing on where the gaps in knowledge and skills are and may be developed into training materials to assist with addressing those gaps.

Leadership from senior management and senior health professionals with commitment to implementation of audit recommendations is essential for the fulfilment of this process. In Tanzania [19] audit teams became disheartened when the same avoidable factors emerged with consecutive maternal deaths because recommended interventions had not been implemented and because of a failure of leadership by senior staff. Health professionals should be accountable to their patients and to the public who have placed their trust in them, to provide a safe environment within which they receive healthcare. The public must see that maternal deaths and other such incidents are treated very seriously, that action is taken to prevent their recurrence. Dialogue and collaboration between stakeholders especially between the Ministry of Health, health facilities and health professional training establishments is essential for supporting changes in clinical practice. Publicising what has been done well is a strong motivator for health professionals and requires more emphasis. In this light the work of the Botswana National Maternal Mortality Audit Committee is applauded and should be further strengthened by inclusion of complementary specialities such as critical care, infectious diseases and HIV management.

## Conclusions

This study shows the interaction between the patient, individual health professional and the health system in generating adverse outcomes for patients. Rather than emphasising individual errors which occur periodically, the causes that lie within the healthcare system and show room for improvement, should be identified, analysed and improved. Root cause analysis is a useful method of identifying factors contributing to maternal deaths, and assists with prioritising interventions with the greatest potential for impact. Training institutions such as nursing and medical schools are well-placed to influence quality of healthcare by implementing education programs that encourage constructive review of the health system as a long-term investment in the health of the population.

## Abbreviations

AIDS: Acquired immunodeficiency syndrome; ANC: Antenatal care; ARV: Antiretroviral; G5P4: Gravida 5 para 4; HIV: Human immune deficiency virus; ICU: Intensive care unit; IDC: Infectious disease care; IRIS: Immune reconstitution inflammatory syndrome; MMR: Maternal Mortality Ratio; MOH: Ministry of Health; NVD: Normal vaginal delivery; PMTCT: Prevention of mother to child transmission; PPH: Postpartum haemorrhage; QI: Quality improvement; RCA: Root cause analysis.

## Competing interests

The authors declare that they have no competing interests.

## Authors’ contributions

FDM, SR and KDM designed the study. FDM, SR, DRM, RP and MH formed the panel that conducted the case reviews. FDM and SR drafted the manuscript and analysed the data. All authors reviewed and commented on drafts of the manuscript. All have seen and approved the final version.

## Pre-publication history

The pre-publication history for this paper can be accessed here:

http://www.biomedcentral.com/1471-2393/14/231/prepub
